# Crude *Ecklonia cava* Flake Extracts Attenuate Inflammation through the Regulation of TLR4 Signaling Pathway in LPS-Induced RAW264.7 Cells

**DOI:** 10.3390/molecules22050777

**Published:** 2017-05-10

**Authors:** Ji-Hyun Hwang, Kui-Jin Kim, Boo-Yong Lee

**Affiliations:** Department of Food Science and Biotechnology, CHA University, Seongnam, Gyeonggi 463-400, Korea; hwangjh1104@naver.com (J.-H.H.); Kuijin.Kim@gmail.com (K.-J.K.)

**Keywords:** *Ecklonia cava* flake extract, inflammation, TLR4, NF-κB, IRF3, MAPKs

## Abstract

We investigated the beneficial effects of the crude *Ecklonia cava* flake (CEF), which is a residual product after polyphenol extraction from *Ecklonia cava*, on inflammation in LPS-stimulated RAW264.7 cells. A group of five different CEF extracts was obtained by a preparation process using water, hydrochloric acid or temperature. We observed that large-size (>19 kDa) CEF extract, which was extracted with water at 95 °C (CEF-W, 95 °C), suppressed the production of inflammatory cytokines by inhibiting its mRNA expression in LPS-induced RAW264.7 cells. TLR4 signaling involvements were negatively regulated by CEF-W, 95 °C. CEF-W, 95 °C repressed the translocation of NF-κB from cytoplasm into nucleus in LPS-induced RAW264.7 cells. CEF-W, 95 °C attenuated the phosphorylation of TBK1 and IRF3 by inhibiting the phosphorylation of ERK. Taken together, we demonstrated that large-size CEF-W, 95 °C may act as a negative regulator of inflammation through the suppression of TLR4 signaling constituents in LPS-induced RAW264.7 cells.

## 1. Introduction

Inflammation is a first defense system to harmful stimuli and causes pain, heat, itching, swelling and redness. Appropriate inflammatory responses are positive effects on the body, excessive responses lead to inflammation related diseases such as cancer, atherosclerosis, rheumatoid arthritis (RA) and asthma [1,2]. The proper regulation of pattern recognition receptors (PPRs) including trans-membrane proteins such as the toll-like receptor (TLRs), c-type lectin receptors (CLRs), and NOD-like receptors (NLRs), have been considered as effective strategies for the reduction of inflammatory responses [3].

In particular, TLRs are the most representative of the PPRs and become activated by different ligands such as double-stranded RNA (dsRNA), flagellin and lipoprotein [4]. TLR4 is binding with gram-negative bacteria such as lipopolysaccharide (LPS) in the cell surface. The production of the inflammatory cytokines, such as tumor necrosis factor superfamily-α (TNF-α), interleukin-6 (IL-6) and interleukin-1β (IL-1β), is induced by TLR4 signaling involvements [5]. In a recent study, macrophages from TLR4-deficient mice did not produce inflammatory cytokines, unlike macrophages from TLR2-deficient mice [6]. It has two different pathways, myeloid differentiation factor 88 (MyD88)-dependent and -independent pathways, unlike the other TLR type [7,8]. MyD88 dependent pathway directly leads to nuclear factor kappa B (NF-κB) activation via the adaptor proteins MyD88 expression. The MyD88 independent pathway promotes the expression of interferon regulatory factor 3 (IRF3) via the activation of the TIR-domain containing adapter inducing interferon β (TRIF) and the TRIF related adaptor molecule (TRAM), which resulted in the translocation of NF-κB and IRF3 into the nucleus.

Mitogen-activated protein kinases (MAPKs) are activated by TLR4 leading to the induction of inflammatory responses. MAPKs include three enzymes, which are p38 MAPK, extracellular signal-regulated kinase (ERK) and c-Jun amino-terminal kinase (JNK) [9]. The p38 MAPK is related to the production of inflammatory cytokines in the pathogenesis of inflammatory diseases [10,11,12]. ERK and JNK also induce inflammation-associated disease progression by regulating the secretion of inflammatory cytokines such as IL-6, IL-10, IL-18, and TNF-α. Furthermore, Ras—a protein modulating to Raf-MEK-ERK—activation provokes local inflammation. Thus, regulation of MAPK pathways appears promising in the field of therapy for the inhibition of inflammatory responses.

Several studies have demonstrated that various phytochemicals and seaweeds have a beneficial effect on oxidative stress, metabolic syndrome, and inflammation [13,14,15]. *Ecklonia cava* (*E. cava*), a brown alga, is a major marine food in the Asia-Pacific region [16]. Recently, it has been reported that *E. cava* derivatives (mainly polyphenols), such as seapolynol and dieckol have radical scavenging activity, anti-adipogenesis, and anti-inflammation [17,18]. However, it is unknown whether the crude *E. cava* flake (CEF), which is a residual product after polyphenol extraction from *E. cava*, has any beneficial effect on anti-inflammation. In the present study, we prepared CEF extraction by using two independent solvents (water and hydrochloric acid) under different temperature conditions to obtain various organic compounds from the original source of the flake. Next, we investigated whether CEF extracts suppress inflammatory responses depending on the methods used for extraction in LPS-induced inflammatory responses in RAW264.7 cells, a murine macrophage cell line, and whether these CEF extracts are regulated to the TLR4 and MAPKs pathways in LPS-stimulated RAW264.7 cells.

## 2. Results

### 2.1. Preparation of the CEF Extracts with Different Conditions

**Figure 1:**
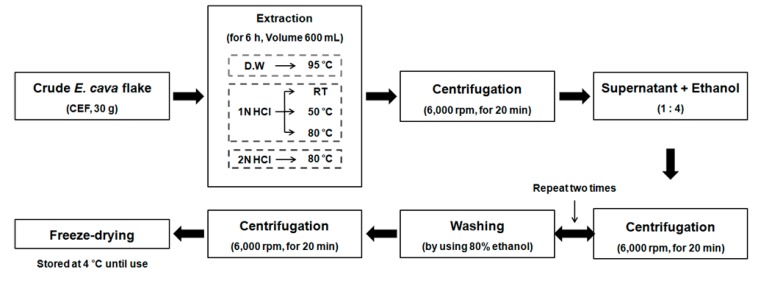
The preparation process of crude *Ecklonia cava* flake (CEF) extracts.

Depending on the extraction solvent and condition, various organic compounds have been derived from the original source of the flake [19]. CEF was extracted with two independent solvents at different temperature conditions including 25 °C, 50 °C, 80 °C, or 95 °C for 6 h (Figure 1). The groups of five different CEF extracts were purified using ethanol and centrifugation, as described in the Materials and Methods section.

As shown in Table 1, the extract yield of CEF extracted with water at 95 °C (CEF-W, 95 °C), CEF extracted with 1 N HCl at RT (CEF-1 N HCl, RT), CEF extracted with 1 N HCl at 50 °C (CEF-1 N HCl, 50 °C), CEF extracted with 1 N HCl at 80 °C (CEF-1 N HCl, 80 °C), and CEF extracted with 2 N HCl at 80 °C (CEF-2 N HCl, 80 °C) were 14.96%, 5.10%, 3.40%, 1.27%, or 0.37%, respectively.

**Figure 2:**
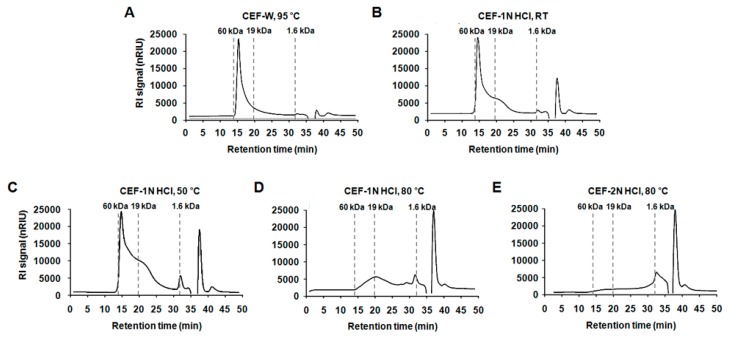
The molecular weight of CEF extracts. (**A**) CEF extract with water at 95 °C; (**B**) CEF extract with 1 N HCl at room temperature (RT); (**C**) CEF extract with 1 N HCl at 50 °C; (**D**) CEF extract with 1 N HCl at 80 °C; and (**E**) CEF extract with 2 N HCl at 80 °C.

Next, high-performance liquid chromatography (HPLC) analysis was conducted to determine the molecular weight of the extracted compounds. As shown in Figure 2 and Table 2, > 19 kDa compounds in CEF-W, 95 °C, CEF-1 N HCl, RT, CEF-1 N HCl, 50 °C, CEF-1 N HCl, 80 °C, and CEF-2 N HCl, 80 °C were observed at approximately 70.46%, 60.66%, 48.15%, 14.30%, or 8.25%, respectively. Less than 1.6 kDa compounds in CEF-W, 95 °C, CEF-1 N HCl, RT, CEF-1 N HCl, 50 °C, CEF-1 N HCl, 80 °C, and CEF-2 N HCl, 80 °C were observed at approximately 10.30%, 16.93%, 19.16%, 47.73%, or 64.97%, respectively. There were no big differences in composition of more than 1.6 kDa and less than 19 kDa compounds among the groups. These data suggested that CEF-W, 95 °C included a large amount of >19 kDa compounds compared to other extraction method applied groups, while CEF-2 N HCl, 80 °C had a large amount of 1.6 kDa > compounds compared to other groups.

### 2.2. Evaluation of Cytotoxic Assay of CEF Extracts on RAW264.7 Cells

**Figure 3:**
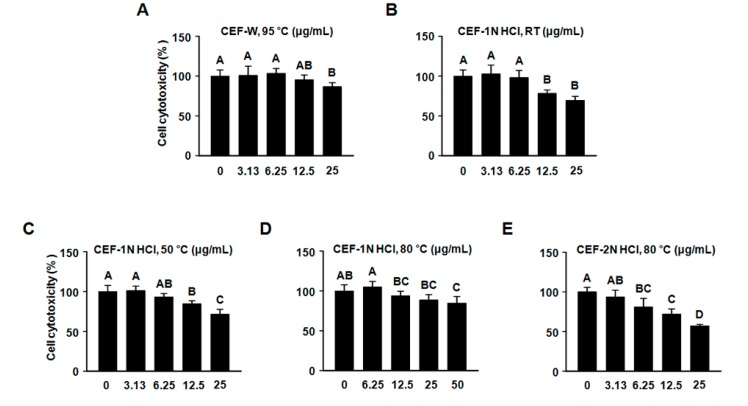
Effect of CEF extracts on cytotoxicity on RAW264.7 cells. Viability of cells treated for 24 h with CEF extracts. Absorbance at 570 nm was recorded in an ELISA plate reader. (**A**) CEF extract with water at 95 °C; (**B**) CEF extract with 1 N HCl at room temperature (RT); (**C**) CEF extract with 1 N HCl at 50 °C; (**D**) CEF extract with 1 N HCl at 80 °C; and (**E**) CEF extract with 2 N HCl at 80 °C. Values with different letters (A–D) are significantly different, *p* < 0.05. The experiment was performed in hexaplicate.

Prior to evaluating the CEF extracts on LPS-induced inflammation in RAW264.7 cells, we performed a cytotoxic assay to select the proper concentration of CEF extracts for further investigation. Cells were incubated with increasing concentrations of CEF extracts (0 μg/mL, 3.13 μg/mL, 6.25 μg/mL, 12.5 μg/mL, 25 μg/mL, and 50 µg/mL) for 24 h. We observed that CEF-1 N HCl, 50 °C, CEF-1 N HCl, 80 °C, and CEF-2 N HCl, 80 °C at 12.5 μg/mL were toxic to cells (Figure 3). CEF-1 N HCl, RT at 25 μg/mL was found to be toxic to cells. CEF-W, 95 °C was not observed at the cytotoxic effect up to 25 μg/mL on cells. Therefore, the overall concentration of 3.13 μg/mL and 6.25 μg/mL CEF-W, 95 °C, CEF-1 N HCl, RT, CEF-1 N HCl, 50 °C, CEF-1 N HCl, 80 °C, and CEF-2 N HCl, 80 °C were selected for further experiments.

### 2.3. Effect of CEF Extracts on the Production of Nitric Oxide (NO) and the Protein Expression of Inducible Nitric Oxide Synthase (iNOS) in LPS-Induced RAW264.7 Cells

**Figure 4:**
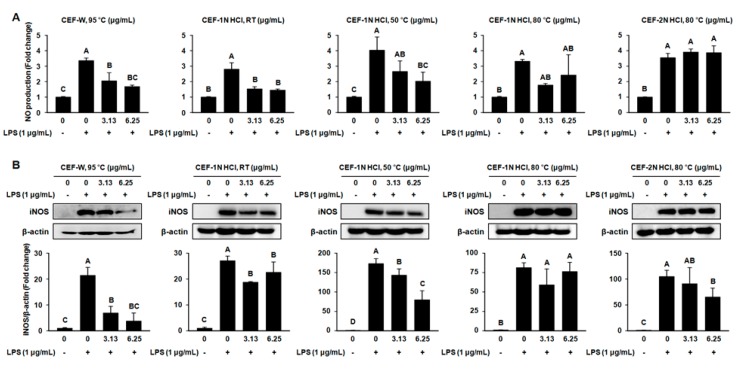
Effect of CEF extracts on nitric oxide (NO) production and the protein expression of iNOS in LPS-induced RAW264.7 cells. RAW264.7 cells were pre-treated with CEF extracts (0 μg/mL, 3.13 μg/mL, and 6.25 μg/mL) for 6 h and then co-treated with LPS (1 μg/mL) for 24 h. (**A**) The production of NO in LPS-induced RAW264.7 cells with the presence or absence of CEF extracts by using Griess reagent at 550 nm; (**B**) The expression of iNOS protein in LPS-induced RAW264.7 cells with the presence or absence of CEF extracts by using western blot. The protein level of iNOS was normalized to the protein level of β-actin. Values with different letters are significantly different, *p* < 0.05. The experiment was performed in triplicate.

To determine the effect of CEF extracts on LPS-induced inflammation, cells were stimulated to inflammation with LPS in the presence or absence of CEF extracts for 24 h. The production of NO is one of the markers of LPS-induced inflammation [20]. As shown in Figure 4A, LPS stimulated the production of NO in RAW264.7 cells, while CEF-W, 95 °C and CEF-1 N HCl, RT significantly suppressed the production of NO. Additionally, CEF-W, 95 °C and CEF-1 N HCl, RT inhibited the expression of iNOS, which is an NO production-associated protein, compared to the LPS-treated cells shown in Figure 4B. Although NO production was slightly inhibited by CEF-1 N HCl, 50 °C, it was not sufficient to attenuate the expression of the iNOS protein. There was no effect on NO production and the expression of iNOS protein in the LPS-induced RAW264.7 cells treated with CEF-1 N HCl, 80 °C or CEF-2 N HCl, 80 °C.

### 2.4. The Production of Pro-Inflammatory Cytokines such as TNF-α, IL-1β, IL-6, and IL-10 Was Suppressed by CEF-W, 95 °C or CEF-1N HCl, RT in LPS-Induced RAW264.7 Cells

**Figure 5:**
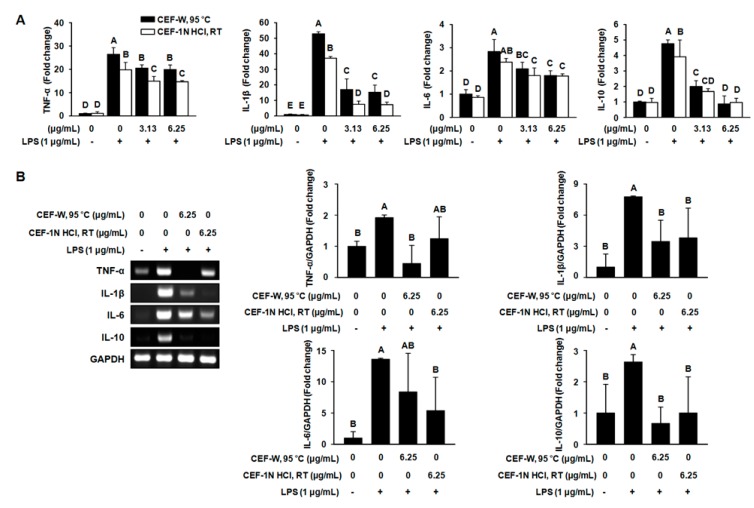
CEF extracts suppress the production of inflammatory cytokines and its mRNA expression in LPS-induced RAW264.7 cells. RAW264.7 cells were pre-treated with CEF-W, 95 °C or CEF-1 N HCl, RT for 6 h and then stimulated for 24 h with LPS (1 μg/mL). (**A**) The production of inflammatory cytokines in supernatants was measured using ELISA kit at 450 nm and 560 nm; (**B**) The mRNA expression of pro-inflammatory cytokines was measured by RT-PCR. The mRNA expression was normalized to the mRNA expression of GAPDH. Values with different letters (A–E) are significantly different, *p* < 0.05. The experiment was performed in triplicate.

It is well known that activated LPS activate the TLR signaling pathway and subsequently produce pro-inflammatory cytokines in macrophage cells. To determine the effect of CEF-W, 95 °C and CEF-1 N HCl, RT on pro-inflammatory cytokines, cells were stimulated with LPS in the presence or absence of CEF-W, 95 °C and CEF-1 N HCl for 24 h. As shown in Figure 5A, it was observed that LPS increased the production of TNF-α, IL-1β, IL-6, and IL-10 when compared to cells treated with the absence of LPS, while CEF-W, 95 °C and CEF-1 N HCl, RT at 3.13 µg/mL and 6.25 µg/mL decreased the LPS-induced production of TNF-α, IL-1β, IL-6, and IL-10 compared to the corresponding control.

Consistent with the production of TNF-α, IL-1β, IL-6, and IL-10, CEF-W, 95 °C and CEF-1 N HCl, RT at 6.25 µg/mL dramatically inhibited the expression of TNF-α, IL-1β, IL-6, and IL-10 mRNA compared to LPS treated groups (Figure 5B). These data indicated that CEF-W, 95 °C and CEF-1 N HCl, RT potentially suppressed the upstream signaling such as TLR-dependent pathways during LPS-stimulated inflammation.

### 2.5. Effect of CEF-W, 95 °C and CEF-1 N HCl, RT on Downstream Target of the TLR Signaling Pathway

**Figure 6:**
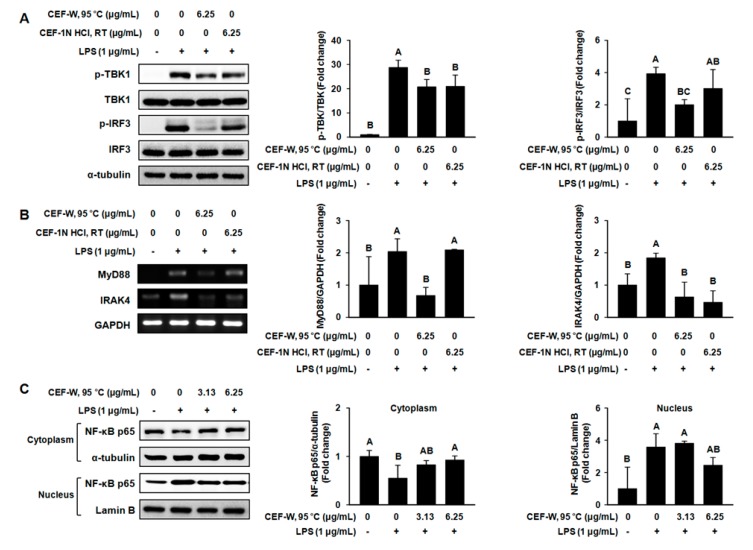
CEF-W, 95 °C and CEF-1 N HCl, RT attenuate the downstream targets of the TLR4 signaling pathway in LPS-induced RAW264.7 cells. RAW264.7 cells were pre-treated with CEF-W, 95 °C and CEF-1 N HCl, RT (0 μg/mL, 3.13 μg/mL, and 6.25 μg/mL) for 6 h, followed by co-treatment with LPS (1 μg/mL) for 30 min. (**A**) The expression of IRF3 and TBK1 proteins was measured using western blot; (**B**) The mRNA levels of MyD88 and IRAK4 was measured by RT-PCR; (**C**) The translocation of NF-κB p65 protein was measured using western blot. The amount of phosphorylated TBK1 and IRF3 was normalized to the amount of total TBK1 protein or total IRF3 protein, respectively. The mRNA expression was normalized to the mRNA expression of GAPDH. The amount of NF-κB p65 protein was normalized to the amount of α-tubulin or lamin B, respectively. Values with different letters (A–C) are significantly different, *p* < 0.05. The experiment was performed in triplicate.

To understand the molecular mechanism underlying the anti-inflammation of CEF-W, 95 °C and CEF-1 N HCl, RT, we investigated the expression of TLR signaling-associated proteins. As shown in Figure 6A, LPS increased the phosphorylation of TBK1 and its downstream target IRF3 phosphorylation, while CEF-W, 95 °C at 6.25 µg/mL suppressed the phosphorylation of TBK1 and IRF3. Moreover, CEF-1 N HCl, RT at 6.25 µg/mL slightly decreased the phosphorylation of TBK1 and IRF3, but was not strong enough when compared to CEF-W, 95 °C. Consistent with western blot analysis, RT-PCR data revealed that the expression of MyD88 and interleukin-1 receptor-associated kinase 4 (IRAK4) mRNA were increased in LPS-induced RAW264.7 cells (Figure 6B). In contrast, CEF-W, 95 °C at 6.25 µg/mL repressed LPS-mediated MyD88 and IRAK4 induction, and CEF-1 N HCl, RT at 6.25 µg/mL showed a decrease tendency in MyD88 and IRAK4 mRNA in LPS-induced RAW264.7 cells when compared with the corresponding control.

The NF-κB p65, suggested that downstream of the regulator of MyD88, TBK1, IRF3, and IRAK4 in the TLR signaling pathway, is a crucial factor to progress LPS-induced inflammation [21]. We further investigated the effect of CEF-W, 95 °C on the translocation of the NF-κB protein that contributed the LPS-mediated inflammation. To evaluate the effect of CEF-W, 95 °C on the translocation of NF-κB p65 from cytoplasm into nucleus, cells were pre-treated with CEF-W, 95 °C at 6.25 µg/mL for 6 h and then incubated with LPS for 30 min. Western blot was performed to determine the translocation of NF-κB p65. As shown in Figure 6C, LPS-stimulated the translocation of NF-κB p65 from cytoplasm into nucleus in cells compared to the corresponding control, while CEF-W, 95 °C, RT reduced the LPS-induced translocation of NF-κB p65 in cells, but not in RAW264.7 treated with CEF-1 N HCl, RT (data not shown). These data suggested that CEF-W, 95 °C had a beneficial effect on reducing the LPS-mediated inflammatory response than CEF-1 N HCl, RT.

### 2.6. CEF-W, 95 °C Attenuates a Part of MAPK Signaling Pathway in LPS-Induced RAW264.7 Cells

**Figure 7:**
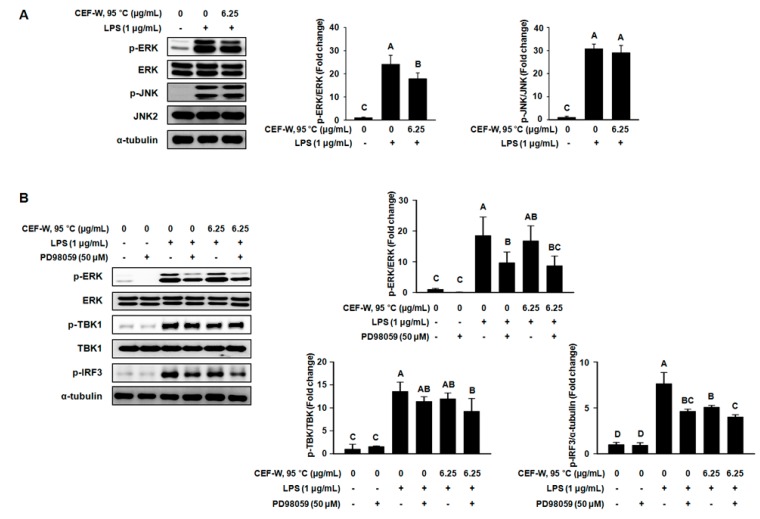
Role of ERK on the anti-inflammatory effect of CEF-W, 95 °C. After antagonist of ERK (PD98059) treatment for 2 h, RAW264.7 cells were pre-treated with CEF-W, 95 °C for 6 h and then stimulated for 30 min with LPS (1 μg/mL). (**A**) The expression of ERK and JNK was measured using western blot; (**B**) Synergistic effect of CEF-W, 95 °C and PD98059 on the LPS-mediated phosphorylation of ERK, TBK1, and IRF3 was measured using western blot. The amount of phosphorylated protein was normalized to the amount of total protein. Values with different letters (A–D) are significantly different, *p* < 0.05. The experiment was performed in triplicate.

Research has shown that MAPKs play an important role in the LPS-induced inflammatory response involving the production of inflammatory cytokines, which can be regulated by antagonist and bioactive compounds [13,22,23,24]. We therefore investigated whether CEF-W, 95 °C regulates MAPKs signaling such as ERK and JNK. As shown in Figure 7A, the phosphorylation of ERK was markedly down-regulated in LPS-induced RAW264.7 cells treated with the presence of CEF-W, 95 °C compared to cells treated with the absence of CEF-W, 95 °C. However, JNK was not affected by CEF-W, 95 °C treatment in LPS-induced RAW264.7 cells. These results indicated that CEF-W, 95 °C might specifically regulate the activity of ERK phosphorylation in LPS-induced RAW264.7 cells.

To investigate whether ERK is targeted by CEF-W, 95 °C, we used an antagonist of ERK (PD98059) in LPS-induced RAW264.7 cells treated with the absence or presence of CEF-W, 95 °C. Consistent with previous results, the phosphorylation of ERK, TBK1, and IRF3 were up-regulated in LPS-induced RAW264.7 cells, while PD98059 dramatically suppressed the phosphorylation of ERK and slightly decreased the phosphorylation in LPS-induced RAW264.7 cells (Figure 7B). Moreover, the combination of CEF-W, 95 °C and PD98059 synergistically suppressed the LPS-mediated phosphorylation of ERK, TBK1, and IRF3 in cells. Thus, our results suggested that CEF-W, 95 °C might inhibit LPS-induced inflammation via regulation of a part of the TLR signaling pathway in an ERK dependent manner.

## 3. Discussion

Inflammation is a very important defense mechanism of the body used to remove harmful stimuli including damaged cells, pathogens, and irritants, and is one of the innate immunity. In particular, acute inflammation is a reaction during infectious challenge and tissue injury, whereas chronic inflammation is a persistent reaction caused by tissue damage [25]. TLR4 plays an important and essential role in the activation of innate immunity [7,26]. Thus, the proper modulation of TLR4 by a synthetic reagent or bioactive compound could be a therapeutic concept to reduce exogenous pathogen-mediated inflammation.

*E. cava* is a brown alga in the Asia-Pacific region [16]. In recently study, has been demonstrated that *E. cava* extracts suppress inflammatory response in LPS-stimulated human endothelial cells [27]. However, it has not indicated whether the CEF has beneficial effect in LPS-induced inflammatory responses. In this study, we compared the beneficial effects of five different compositions of product isolated from CEF, which consisted of 1.6 kDa >, between 1.6 kDa and 19 kDa, or > 19 kDa. Although CEF-1 N HCl, 80 °C and CEF-2 N HCl, 80 °C showed a poor yield compared to others, we speculated that CEF-1 N HCl, 80 °C and CEF-2 N HCl, 80 °C may have beneficial effects of anti-inflammation due to the small size CEF extracts.

The production of NO is a major response to inflammation in macrophage [28]. In general, large-size CEF extracts are relatively difficult to bind with the extra-cellular biological domain when compared to the breakdown of CEF. However, our results revealed that small size CEF extracts including CEF-1 N HCl, 80 °C, CEF-2 N HCl, 80 °C, and CEF-1 N HCl, 50 °C have not shown to inhibit the production of NO and its regulatory protein iNOS in LPS-induced RAW264.7 cells. In contrast, large-size CEF extracts such as CEF-W, 95 °C or CEF-1 N HCl, RT suppressed the production of NO in LPS-induced RAW264.7 cells. Moreover, CEF-W, 95 °C and CEF-1 N HCl, RT also decreased the expression of iNOS protein when compared to the corresponding control. These results indicate that large-size CEF extracts CEF-W, 95 °C and CEF-1 N HCl, RT have beneficial effects of anti-inflammatory activities.

Exogenous pathogens activate the TLRs signaling pathway constituents such as TLR4, MyD88, TBK1, IRF3, and IRAK4, and subsequently stimulate the release of pro-inflammatory cytokines which promote to progress a disease by producing fever and tissue damage [5,29,30,31]. TNF-α, IL-1β, IL-6, and IL-10 are members of a pro-inflammatory cytokine that activate innate immune cells and play a crucial role in exacerbating the inflammatory response [7,32,33,34].

In the present study, we observed that CEF-W, 95 °C and CEF-1 N HCl, RT inhibited LPS-mediated the production of TNF-α, IL-1β, IL-6, and IL-10 and the expression of TNF-α, IL-1β, IL-6, and IL-10 in RAW264.7 cells. The production of these pro-inflammatory cytokines was also regulated by TLR4 signaling in macrophage cells [35]. Therefore, we determined whether TLR4 signaling involvements including MyD88, IRAK4, TBK1, and IRF3 were necessary for the stimulation of pro-inflammatory cytokines. To evaluate the inhibitory effect of CEF-W, 95 °C and CEF-1 N HCl, RT on TLR4 signaling, we used LPS to activate TLR4 signaling in RAW264.7 after pre-incubation of CEF-W, 95 °C and CEF-1 N HCl, RT for 6 h. CEF-1 N HCl, RT slightly suppressed the phosphorylation of TBK1 and IRF3 and the expression of MyD88 and IRAK4 mRNA in LPS-induced RAW264.7 cells, while CEF-W, 95 °C strongly inhibited LPS-mediated phosphorylation of TBK1 and IRF3 in RAW264.7 cells. RT-PCR analysis also revealed that LPS-induced MyD88 and IRAK4 mRNA expression were attenuated by CEF-W, 95 °C.

NF-κB, a downstream target of the TLR4 signaling pathway, acts as a transcriptional regulator of a pro-inflammatory gene. NF-κB p65 subunit dissociates from IκB-α and translocates from the cytoplasm into the nucleus in LPS-induced macrophage cells [13]. In addition, the MAPKs signaling pathway, comprising of p38 MAPK, ERK, and JNK, is required for NF-κB subunit p65 transactivation in LPS-induced inflammatory response [22,35]. Therefore, we examined whether CEF-W, 95 °C affected the translocation of NF-κB p65 subunit in LPS-induced RAW264.7 cells. The results showed that CEF-W, 95 °C repressed the translocation of NF-κB p65 from the cytoplasm into the nucleus compared to LPS-induced RAW264.7 cells. In addition, CEF-W, 95 °C specifically attenuated the phosphorylation of ERK in LPS-induced RAW264.7 cells. Our results also revealed that CEF-W, 95 °C and PD98059 synergistically suppressed the phosphorylation of TBK1, IRF3, and ERK compared to other groups in LPS-induced RAW264.7 cells.

## 4. Materials and Methods

### 4.1. Materials and Reagents

Crude *E. cava* flake (CEF) was obtained from MilaeML (St. Ogeum, Seoul, Korea). RAW264.7 cells were purchased from American Type Culture Collection (Manassas, VA, USA). Dulbecco’s Modified Eagle’s medium (DMEM), Fetal bovine serum (FBS), 100 units/mL penicillin, and 100 μg/mL streptomycin were purchased from Gibco (Grand Island, NY, USA). LPS (*Escherichia coli* 0111:B4) was purchased from Sigma (St. Louis, MO, USA). Phosphate-buffered saline (PBS) was purchased from RMS Bio-solution (Seoul, Korea). Thiazolyl Blue tetrazolium bromide (MTT) was purchased from Alfa Aesar (Ward Hill, MA, USA). Griess reagent was purchased from Sigma (St. Louis, MO, USA). Enzyme-linked immunosorbent assay (ELISA) kits were purchased from BioLegend (San Diego, CA, USA). TRIzol reagent was purchased from Invitrogen (Carlsbad, CA, USA). PD098059 were purchased from Cayman Chemical (Ann Arbor, MI, USA). Primers were purchased from Bioneer (Daejeon, Korea). Antibodies were purchased from Cell Signaling Technology (Boston, MA, USA) and Santa Cruz Biotechnology (Santa Cruz, CA, USA).

### 4.2. Preparation Process of the CEF Extracts

After hot air drying leaf of *E. cava*, it was homogenized and mixed with 30% fermentation alcohol. The mixture was reacted at 80 °C for 1 h and filtered twice. The CEF was the residue remaining after filtration, except for polyphenol.

Dried CEF (30 g) was extracted with water or hydrochloric acid (HCl, 1 N or 2 N) at various temperatures (RT, 50 °C, 80 °C, or 95 °C) for 6 h, respectively. Next, the extracts were centrifuged at 6000 rpm for 20 min and the supernatant was transferred to a new bottle. The supernatants were homogeneous with ethanol and were centrifuged at 6000 rpm for 20 min. After removing the supernatant, the pellet was washed twice in 80% ethanol and centrifuged at 6,000 rpm for 20 min. Samples were freeze-dried in −45 °C under vacuum for at least 48 h and were stored at 4 °C until use.

### 4.3. Analysis of the Molecular Weight of CEF Extracts

The molecular weight of the CEF extracts was determined by high performance-size exclusion chromatography (HPSEC) using an Agilent 1260 Infinity high performance liquid chromatography system equipped with Superdex 75 10/300 GL packed column (10 mm × 300 mm; GE Healthcare, Piscataway, NJ, USA) with 50 mM ammonium formate buffer (pH 5.5) as an eluent at a flow rate of 0.5 mL/min.

### 4.4. Cell Culture and Viability

RAW264.7 cells, a murine macrophage cell line, were grown at 37 °C in a 5% CO_2_ incubation. To evaluate cell viability, RAW264.7 cells (5 × 10^3^ cells/well) were cultured in a 96-well plate. Cells were exposed to the presence and absence of CEF extracts for 24 h. After reacting with the CEF extract, cell viability assay was performed by MTT assay.

### 4.5. Measurement of the Contents of Nitric Oxide

RAW264.7 cells were seeded into six-well plates at a density of 1 × 10^6^ cells/well. RAW264.7 cells were treated with CEF extracts (0 μg/mL, 3.13 μg/mL, and 6.25 μg/mL) and incubated for 6 h. LPS (1 μg/mL) was treated and incubated for 24 h at 37 °C in a CO_2_ incubator. After various treatments, cell culture medium (100 μL) was mixed with Griess reagent (100 μL) and incubated at room temperature for 20 min. The NO concentration was determined at 540 nm using NaNO_2_ as a standard.

### 4.6. Evaluation of the Inflammatory Cytokines Production in RAW264.7 Cells

RAW264.7 cells were pre-treated with CEF extracts for 6 h and then stimulated with or without LPS (1 μg/mL) for 24 h. Supernatants were thawed only once, immediately before performing the cytokine assay. We measured cytokine levels (TNF-α, IL-1β, IL-6, and IL-10) in supernatants using ELISA kits as per the manufacturer’s instructions.

### 4.7. RNA Isolation and Reverse Transcription Polymerase Chain Reaction (RT-PCR)

Total RNAs were extracted using TRIzol reagent and subsequently used to generate cDNA using an RT-PCR system. Target gene amplification was performed using specific oligonucleotide primers in a normal PCR system. The primer sequences were as follows: TNF-α, forward (5′-CTACTCCTCAGAGCCCCCAG-3′) and reverse (5′-TGACCACTCTCCCTTTGCAG-3′); IL-1β, forward (5′-CAGGATGAGGACATGAGCACC-3′) and reverse (5′-CTCTGCACACTCAAACTCCAC-3′); IL-6, forward (5′-CCATCTCTCCGTCTCTCACC-3′) and reverse (5′-AGACCGCTGCCTGTCTAAAA-3′); IL-10, forward (5′-CAGTACAGCCGGGAAGACAA-3′) and reverse (5′-TCCAGCTGGTCCTTTGTTTG-3′); MyD88, forward (5′-ACTGGCCTGAGCAACTAGGA-3′) and reverse (5′-CGTGCCACTACCTGTAGCAA-3′); IRAK4, forward (5′-AGCTGCGTCACCTACCTGTT-3′) and reverse (5′-GTTTGGTGATGTTGCTGTGG-3′); GAPDH, forward (5′-AACTTTGGCATTGTGGAAGG-3′) and reverse (5′-ACACATTGGGGGTAGGAACA-3′). PCR products were analyzed on 1.5% agarose gels and bands were visualized using ethidium bromide staining. The results were quantified using the ImageJ software.

### 4.8. Western Blot Analysis

After RAW264.7 cells were washed using 1 × PBS, cells were lysed by lysis buffer (add as phosphatase inhibitor cocktail 2, phosphatase inhibitor cocktail 3). Cytosolic and nuclear proteins were extracted using NE-PERTM nuclear and cytoplasmic extraction reagents (Thermo Scientific) as per the manual. The protein content was determined using the Bradford assay. Protein extracts were separated by sodium dodecyl sulfate polyacrylamide gel electrophoresis (SDS-PAGE) and transferred to polyvinylidene difluoride (PVDF) membranes (Immune-Blot PVDF membrane, Bio-Rad). Membranes were immunoblotted with primary antibodies specific for iNOS, p-TBK1, TBK1, p-IRF3, IRF3, NF-κB p65, Lamin B, p-ERK, ERK, p-JNK, JNK2, and α-tubulin at 4 °C overnight. Membranes were then treated with horseradish peroxidase (HRP)-conjugated secondary antibodies (1:5000) for 2 h. Bands were visualized using an enhanced chemiluminescence system (ECL, Thermo Fisher Scientific, Waltham, MA, USA) and LAS image software (Fuji, New York, NY, USA). The results were quantified using the ImageJ software.

### 4.9. Statistical Analysis

All experiments were performed in triplicate or hexaplicate. Differences among multiple groups were determined by one-way analysis of variance (ANOVA), followed by Duncan’s multiple range test, using the SPSS software system (SPSS for Windows, version 20; SPSS, Inc., Chicago, IL, USA). Values with different letters are significantly different, *p* < 0.05.

## 5. Conclusions

In conclusion, we demonstrated the anti-inflammatory effect of large-sized CEF extract (CEF-W, 95 °C) on LPS-induced inflammation in macrophage cells through the regulation of the inflammatory signaling pathway, particularly those affecting the suppression of inflammatory cytokines and the inhibition of a part of the TLR4 signaling and MAPK signaling pathways. Based on our findings, we suggest that seaweed-derived crude, particularly large-size (>19 kDa) CEF extracts such as CEF-W, 95 °C, may be a potential ingredient against exogenous pathogen-mediated inflammation.

## Figures and Tables

**Table 1:** The extraction yield of CEF extracts.

Compound	Solvent	Temp.	Recovery (g/30 g)	Yield (%)
CEF-W, 95 °C	D.W	95 °C	4.49	14.96
CEF-1 N HCl, RT	1 N HCl	RT	1.53	5.10
CEF-1 N HCl, 50 °C		50 °C	1.02	3.40
CEF-1 N HCl, 80 °C		80 °C	0.38	1.27
CEF-2 N HCl, 80 °C	2 N HCl	80 °C	0.11	0.37

**Table 2:** The molecular weight analysis of CEF extracts.

Compound	Solvent	Temp.	X > 19 kDa (%)	19 kDa > X > 1.6 kDa (%)	1.6 kDa > X (%)
CEF-W, 95 °C	D.W	95 °C	70.46 ± 3.28 ^A^	19.24 ± 1.19 ^D^	10.30 ± 1.68 ^D^
CEF-1 N HCl, RT	1 N HCl	RT	60.66 ± 1.67 ^B^	22.40 ± 1.73 ^C,D^	16.93 ± 3.40 ^C^
CEF-1 N HCl, 50 °C		50 °C	48.15 ± 0.90 ^C^	32.69 ± 2.34 ^B^	19.16 ± 0.23 ^C^
CEF-1 N HCl, 80 °C		80 °C	14.30 ± 1.29 ^D^	37.98 ± 3.15 ^A^	47.73 ± 0.55 ^B^
CEF-2 N HCl, 80 °C	2 N HCl	80 °C	8.25 ± 1.21 ^E^	26.78 ± 5.82 ^C^	64.97 ± 4.54 ^A^

Values with different letters (A–E) are significantly different, *p* < 0.05.
